# TENDINOPATHY AND OBESITY

**DOI:** 10.1590/0102-6720201600S10026

**Published:** 2016

**Authors:** Adham do Amaral e CASTRO, Thelma Larocca SKARE, Paulo Afonso Nunes NASSIF, Alexandre Kaue SAKUMA, Wagner Haese BARROS

**Affiliations:** Postgraduate Program in Principles of Surgery, Evangelic Faculty of Paraná/University Evangelic Hospital of Curitiba/Medical Research Institute, Curitiba, PR, Brazil

**Keywords:** Tendinopathy, Obesity, Overweight, Adipokines

## Abstract

**Introduction:**

Tendinopathies and tendon tears account for over 30% of all musculoskeletal consultations. Obesity, which is becoming one of the world´s most prevalent public health issues, may be associated with this condition.

**Objective:**

To review the literature about tendinopathies and obesity association.

**Methods:**

This is a descriptive exploratory study using the portal Medline. Literature in English language from 2006 to 2014 were reviewed.

**Results:**

The pathogenesis of tendinopathies includes inflammatory, regenerative and degenerative processes that happen simultaneously from early to late phases of the disease. Mechanical stress upon tendons seems to be one of the most important factors to initiate the inflammatory response, but it´s not the only one that can deflagrate it: there are other extrinsic, genetic and metabolic factors that may be involved. Therefore, tendinopathies in obese patients can be due to tendon overload because of the excess of weight, but also because of increased production of pro-inflammatory mediators related to fat tissue such as adipokines. This pro-inflammatory state that obese people can suffer is known as adiposopathy, or sick fat syndrome. Weight loss is associated with decrease in adipokines and improvement of musculoskeletal symptoms.

**Conclusion:**

The relation of obesity and tendinopathies is supported by evidences of recent studies, exemplified in this review of literature.

## RESUMO

**Introdução:**

As tendinopatias e as fissuras em tendões respondem por 30% de todas as consultas médicas. A obesidade, que está se tornando um dos problemas de saúde pública mais prevalentes no mundo, pode estar associada com esta condição.

**Objetivo:**

Revisar a literatura acerca da associação entre obesidade e tendinopatias.

**Métodos:**

Este é um estudo exploratório e descritivo utilizando artigos em língua inglesa do portal médico Medline, do período de 2006 a 2014.

**Resultados:**

Na patogênese das tendinopatias incluem-se elementos inflamatórios, regenerativos e degenerativos que aparecem de maneira simultânea em todos os estágios da doença. O estresse mecânico sobre os tendões parece ser um dos mais importantes na promoção do processo inflamatório inicial. Todavia não é o único. Existem fatores ambientais, genéticos e metabólicos atuando de maneira ativa. Portanto, as tendinopatias em indivíduos obesos podem se dever à sobrecarga mecânica pelo excesso de peso, mas, também, pelo aumento na produção de mediadores pró-inflamatórios relacionados ao tecido adiposo, como as adipocinas. O estado pró-inflamatório existente no indivíduo obeso é conhecido como adiposopatia ou síndrome da "gordura doente". A perda de peso está associada com decréscimo das adipocinas e diminuição da sintomatologia musculoesquelética.

**Conclusão:**

A associação da obesidade com tendinopatias tem sido fundamentada em estudos recentes como os desta revisão de literatura.

## INTRODUCTION

Tendinopathies and tendon tears are very common in medical practice, accounting for over 30% of all musculoskeletal consultations^6^. In this involvement, the tendinous portion of musculotendinous units loses the normal collagenous architecture that is replaced by an amorphous mucinous material^28^. It is hypothesized that an increase in the amount and duration of mechanical load supported by the tendon unleash programmed cell death or apoptosis^28^. 

Tendinopathies affect the physical functioning; cause pain and suffering. They may have economic implications for the patients with a negative impact in their quality of life^4^ ^,^ ^12^ ^,^ ^30^ ^,^. The prompt and accurate diagnosis is important for the correct treatment avoiding chronicity and disability^42^. The most vulnerable tendons are the Achilles, patellar, the rotator cuff and extensor carpis radialis brevis tendons^4^.

There are two main types of tendon diseases: the enthesopathy and the tendinopathy. In the first, the inflammatory and mechanical injuries occur at the junction of tendon with the bone; in the second in the tendon midportion. These two types must be differentiated as they may have different etiologies^4^. 

 It has been shown that obesity may be associated with tendinopathies. Besides the well-established health problems related to obesity such as vascular and heart diseases, the musculoskeletal implications of overweight have been more and more studied due to their huge economic burden. Obesity is becoming one of the most prevalent public health issues in the whole world ^3^ ^,^ ^8^ ^,^ ^14^ ^,^ ^16^ ^,^ ^17^ ^,^ ^19^ ^,^ ^20^ ^,^ ^32^ ^,^ ^39^ ^,^ ^46^ ^,^ ^47^ ^,^ ^50^ ^-^ ^53^. The 2015 World Health Organization projection showed that 2.3 billion adults are overweight and more than 700 million are obese^52^. This highlights the importance of studying all interferences of obesity in daily life.

 The objective of this study was to do a literature review on the relationship of obesity and tendinopathies, first, focusing on clinical evidences and them focusing on its pathophysiology.

## METHODS

This study is a review of literature using the Pubmed Database. In October, 2015, it was accessed the portal using the following descriptors in English: "obesity"," overweight" and "body mass index" combined with the descriptors in English "tendinopathy", "tendinitis", "rotator cuff", "epicondylitis", "wrist", "patellar", "quadriceps", "Achilles", "plantar fascia" and "tendon". 

The target studies of this review were the ones that had as the main objective or one of the main objectives the verification of the relationship between obesity or excess of weight and any kind of tendinopathy. After excluding repeated papers and considering only the clinical studies, the search resulted in 59 papers. But 49 of them were than excluded for the following reasons: studies that verified the relation of any tendinopathy with BMI, but in athletes instead of overweight or obese people; studies that did not made a statistic evaluation of BMI with tendinopathy and showed only frequency; study that evaluated BMI just as a marginal result of a tendinopathy, not being the main or one of the main objectives; studies that evaluated tendon tears instead of tendinopathy. After this process there were 10 articles, which formed the basis of this paper.

The 10 articles were classified according to the following variables: authors, year of publication, study design, number of patients, measure of tendinopathy, results and conclusions.

## RESULTS

Firstly, was sought the name of the first author and year of publication. Then, the study design was analyzed, number of patients, measure of tendinopathy and finally their results and conclusions.

 All the 10 articles were published in English and the time of publication varied from 2006 to 2014. The tendinopathies analyzed were: rotator cuff, pattelar, medial and lateral epicondylitis, Achilles, trigger finger, posterior tibial, peroneal tendons, plantar fascia and pes anserinus. The type of studies was case control and cross sectional (some of them involving population). 

**FIGURE 1:**
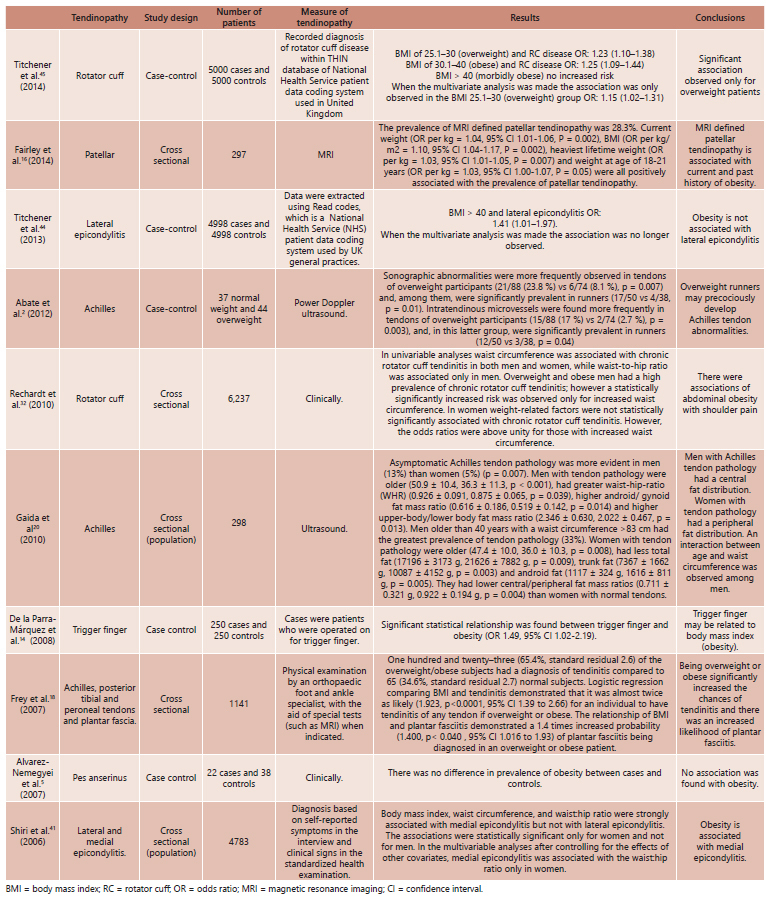
Distribution of the 10 analyzed articles and their characteristics

Figure 1 shows the 10 studies characteristics.

## DISCUSSION

Obesity is a metabolic disease that has grown rapidly globally. The brake to this growth is one of the goals of the World Health Organization (WHO) to be achieved by 2025. WHO estimates in its latest data that more than one third of adults older than 18 years are now overweight, being 38% men and 40% women. In addition, it is estimated that the worldwide prevalence of obesity doubled between 1980 and 2014, being obese in 2014, 11% of men and 15% women, accounting for more than half a billion obese adults worldwide^53^.

Tendinopathy is the most prevalent tendon disorder, and various preventive interventions have been investigated. Cook and Purdam (2009)^11^ have proposed a model of tendinopathies, involving histological, clinical and imaging information aiming to understand their different presentation. In their model, characterized as a continuum of alterations, the tendinopathy starts as a result of an insult (generally an acute overload or compression) with a non-inflammatory proliferative tissue reaction and minimal collagen damage. In the following stage, the collagen splits up and abnormal tenocytes proliferation with neovascularization occurs. If the patient is treated in these two initial stages, the process may be reversible. In the last stage of this model, there is accentuated disruption of collagen fibers, diffuse cell death with appearance of neo-vessels and nerves in the substance of the tendon^11^. At this level the situation is irreversible. These three phases has been denominated as: 1) reactive tendinopathy; 2) tendon disrepair; and 3) degenerative tendinopathy^26^.

 Mc Creesh and Lewis (2013)^26^ studying the pathogenic process of tendinopathies noted that inflammation and degeneration frequently occurs simultaneously. They defined that inflammatory, regenerative and degenerative processes happen in all stages, from early to late phases. The role of the inflammatory process in this context is not totally clear and may vary depending of which tendon is affected^24^.

 Mechanical stress upon tendons may be one of the main factors involved in the appearance of tendinopathies^26^. Healthy tendons have elastic properties being able to adapt to the tensions through changes in their mechanical properties and structure^26^. Overload or repetitive use may result in tendon disease and seems to be the dominant factor initiating the inflammatory response. It is believed that some of the damage caused by the overload is mediated through inflammatory process^2^ ^,^ ^25^ ^,^ ^33^ ^,^ ^38^.

In addition to loading exposure, a great number of others extrinsic and intrinsic factors may interfere with development of tendinopathy^26^. Anatomical features, posture, occupational and sporting activities are found among the extrinsic factors. Some individuals may have a genetic predisposition^1^. Likewise, metabolic features play a role in tendinopathy, with diabetes mellitus being a well-known risk factor since long time ago^1^. Obesity is not so valued in this context, although this concept is changing as the knowledge in this field is growing. 

So, tendinopathies in obese patients may be due not only to joint and tendon overload, but also because of increased production of pro-inflammatory mediators^7^ ^,^ ^15^ ^,^ ^17^ ^,^ ^40^.

Adipose tissue is now recognized as a multifunctional organ. It plays an important role as an energy storage organ, but it also releases active pro- inflammatory molecules such as IL-6, TNF-α, and leptin that act on immune cells leading to local and systemic inflammation^27^. The inflammatory mediators elaborated in the fat tissue are generated by local macrophages that are increased in number in obese people; the percentage of these cells in adipose tissue ranges from less than 10% in lean individuals to 40-50% in the obese^30^. The inflammatory cytokines produced by adipocytes acts recruiting more macrophage, therefore perpetuating a vicious cycle of inflammation^27^.

Not all obese patients suffer from chronic inflammation; there is a group of patients where the metabolic inflammatory syndrome predominates. Bays (2014)^7^ defined adiposopathy, also known as sick fat syndrome, as the result of "a pathologic adipose tissue anatomic/functional disturbances promoted by positive caloric balance in genetically and environmentally susceptible individuals which results in adverse endocrine and immune responses that both directly and indirectly contribute to metabolic disease and increased cardiovascular disease risk"^7^. These people have increased rates of cancer, asthma, atherosclerosis, rheumatoid arthritis, diabetes, osteoporosis, Alzheimer's disease, osteoarthritis and depression^27^. Adiposopathy also leads to pain chronicity because the related non-resolving systemic inflammation that causes a pathophysiologic state that promotes nociception in dysfunctional musculoskeletal tissues and avoids healing and pain resolution^27^ ^,^ ^40^.

 Some studies have shown that overweight and obesity are related with disability affecting basic activities of daily living^22^ ^,^ ^34^; it also increases risk of chronic diseases with their secondary symptoms^23^. All of this impacts people´s quality of life. More recent studies have focused in quality of life changes before and after weight loss^10^ ^,^ ^21^ ^,^ ^31^ ^,^ ^35^ ^,^ ^36^ ^,^ ^39^ ^,^ ^40^ ^,^ ^43^ ^,^ ^48^ ^,^ ^49^ ^,^ ^53^. Weight loss is capable of improve quality of life, and to reduce risks of obesity associated diseases^43^, even if the weight loss is modest^36^.

Linkov et al. (2014)^25^ demonstrated that the weight loss was associated not only with better quality of life, but also with decrease in adipokines; they also demonstrated a correlation between the level adipokines reduction and improvement of the physical quality of life^25^.

Birn et al. (2015)^8^ studied the association between musculoskeletal symptoms of obese patients before and after bariatric surgery. They found that patients with musculoskeletal disorders before the surgery experienced a larger improvement of quality of life after weight reduction than participants without symptoms before the procedure^8^. These results indicate an important role of musculoskeletal symptoms in the quality of life of obese people.

Bout-Tabaku et al. (2015)^10^ demonstrated that adolescents with severe obesity have musculoskeletal pain that limits their quality of life. They also demonstrated that c-reactive protein (CRP) levels were associated with higher BMI values and with musculoskeletal pain in crude analysis; but after adjustment, no significant association was observed between pain and CRP, suggesting that pain mechanisms of nociceptor activation are complex and related to cytokines, but it is not specific to CRP^10^.

## CONCLUSION

Obesity is becoming one of the most important world´s health issues. Adding this to the facts that tendinopathies are one of the most frequent causes of musculoskeletal medical consultations and that obesity may be one of its cause, the scenario of the obese patient in the orthopedic doctors' office tends to become more frequent nowadays. So, it is important to be aware of the reviewed evidences that support the association between these two diseases to better manage these patients.
